# Supplementary material for: Evolutionary origins and life-history correlates of coloniality in the epiphytic fern genus Platycerium (Polypodiaceae)
Source: PLoS One. 2025 Aug 1;20(8):e0329099. doi: 10.1371/journal.pone.0329099 (PMC12316288; doi:10.1371/journal.pone.0329099)
Supplement: S1 File — (DOCX) [file pone.0329099.s001.docx]

**Table S1.** Results of Bayesian mixed effect models to assess significant relationship between the response variable height and the dependent variables strap frond angle (SFAn) and nest frond area (NFAr). The estimate (positive or negative) relationship can be considered significant if its 95% confidence interval does not span zero. Significant associations are indicated with **. Rhat, Bulk ESS and Tail ESS are measures used to assess the quality and reliability of the posterior distribution in a Bayesian model. Rhat measures the convergence of the Markov Chain Monte Carlo (MCMC) sampling, and should always be lower than 1.02. Bulk ESS measures effective sample size and accounts for autocorrelation within the MCMC chain. Large Bulk ESS values (> 1000) indicate that the chains have satisfactorily explored the posterior distribution, leading to reliable estimates of the mean. Tail ESS measures effective sample size in the tail regions of the distribution. Large Tail ESS values (> 1000) indicate that the model has explored well the extreme ends of the distribution, leading to reliable estimates of the confidence intervals.

| **Species** | **Dependent variable** | **Mean Estimate** | **Std. Error** | **95% CI (Lower)** | **95% CI (Upper)** | **Rhat** | **Bulk ESS** | **Tail ESS** |
| --- | --- | --- | --- | --- | --- | --- | --- | --- |
| *P. alcicorne* | SFAn | 0.06** | 0.02 | 0.03 | 0.09 | 1.00 | 1106 | 1171 |
|  | NFAr | 14.57 | 10.76 | -7.44 | 36.39 | 1.00 | 2202 | 1889 |
| *P. andinum* | SFAn | -0.02 | 0.04 | -0.09 | 0.06 | 1.01 | 2037 | 1183 |
|  | NFAr | 464.72 | 198.3 | -1.56 | 709.27 | 1.06 | 600 | 142 |
| *P. angolense* | SFAn | -0.04 | 0.03 | -0.10 | 0.02 | 1.00 | 1141 | 1578 |
|  | NFAr | 40.86** | 6.41 | 29.67 | 57.25 | 1.00 | 1450 | 724 |
| *P. bifurcatum* | SFAn | 0.15** | 0.03 | 0.09 | 0.21 | 1.00 | 1703 | 1757 |
|  | NFAr | 108.92** | 36.74 | 36.76 | 180.64 | 1.00 | 1890 | 1918 |
| *P. coronarium* | SFAn | -0.03 | 0.03 | -0.09 | 0.03 | 1.00 | 1347 | 1681 |
|  | NFAr | 14.41 | 65.38 | -122.20 | 135.56 | 1.00 | 1474 | 1810 |
| *P. ellisii* | SFAn | 0.21 | 0.19 | -0.19 | 0.61 | 1.00 | 1437 | 1130 |
|  | NFAr | 19.32 | 13.89 | -9.22 | 49.17 | 1.00 | 1140 | 1050 |
| *P. hillii* | SFAn | 0.06 | 0.04 | -0.01 | 0.12 | 1.00 | 979 | 1333 |
|  | NFAr | 32.73** | 15.96 | 0.51 | 63.75 | 1.00 | 1455 | 1960 |
| *P. madagascariense* | SFAn | 0.03 | 0.15 | -0.25 | 0.33 | 1.00 | 1735 | 1232 |
|  | NFAr | -7.89 | 19.81 | -49.81 | 30.27 | 1.00 | 1824 | 1328 |
| *P. quadridichotomum* | SFAn | 0.01 | 0.03 | -0.04 | 0.06 | 1.00 | 1725 | 1856 |
|  | NFAr | 125.65** | 23.66 | 76.81 | 172.80 | 1.00 | 2459 | 2110 |
| *P. stemaria* | SFAn | 0.06 | 0.03 | -0.01 | 0.12 | 1.01 | 768 | 1203 |
|  | NFAr | 32.64** | 16.43 | 0.68 | 66.23 | 1.00 | 1630 | 1788 |
| *P. veitchii* | SFAn | 0.06 | 0.03 | -0.01 | 0.12 | 1.00 | 782 | 1068 |
|  | NFAr | 32.40** | 16.43 | 0.02 | 65.52 | 1.00 | 1554 | 2006 |
| *P. willinckii* | SFAn | 0.69** | 0.28 | 0.14 | 1.22 | 1.00 | 1328 | 1942 |
|  | NFAr | 72.32** | 24.03 | 18.03 | 116.61 | 1.00 | 722 | 1054 |

**Table S2.** Results of discrete ancestral state reconstruction on coloniality. Each species (tree tip) was categorised as belonging to 1 of 3 possible states, being either solitary, colonial with monomorphic colony members or colonial with polymorphic colony members. Nodes 27 to 51 represent all internal nodes, and numbers for each state represent the probability for that node to be in that state. For the position of each internal node see Fig S3.

| **Species/Node** | **Solitary** | **Colonial, monomorphic** | **Colonial, polymorphic** |
| --- | --- | --- | --- |
| Pyrrosia_bonii | 1 | 0 | 0 |
| Pyrrosia_subfurfuracea | 1 | 0 | 0 |
| Pyrrosia_assimilis | 1 | 0 | 0 |
| Pyrrosia_lingua | 1 | 0 | 0 |
| Pyrrosia_petiolosa | 1 | 0 | 0 |
| Pyrrosia_angustissima | 1 | 0 | 0 |
| Hovenkampia_schimperiana | 1 | 0 | 0 |
| Platycerium_andinum | 0 | 1 | 0 |
| Platycerium_stemaria | 0 | 0 | 1 |
| Platycerium_angolense | 0 | 0 | 1 |
| Platycerium_ellisii | 0 | 1 | 0 |
| Platycerium_alcicorne | 0 | 0 | 1 |
| Platycerium_madagascariense | 0 | 1 | 0 |
| Platycerium_quadridichotomum | 0 | 0 | 1 |
| Platycerium_holttumii | 1 | 0 | 0 |
| Platycerium_grande | 1 | 0 | 0 |
| Platycerium_wandae | 1 | 0 | 0 |
| Platycerium_superbum | 1 | 0 | 0 |
| Platycerium_wallichii | 1 | 0 | 0 |
| Platycerium_coronarium | 0 | 1 | 0 |
| Platycerium_ridleyi | 1 | 0 | 0 |
| Platycerium_veitchii | 0 | 0 | 1 |
| Platycerium_willinckii | 0 | 0 | 1 |
| Platycerium_bifurcatum | 0 | 0 | 1 |
| Platycerium_hillii | 0 | 0 | 1 |
| Thylacopteris_papillosa | 1 | 0 | 0 |
| 27 | 0.861 | 0.133 | 0.006 |
| 28 | 0.879 | 0.116 | 0.005 |
| 29 | 0.993 | 0.007 | 0 |
| 30 | 0.994 | 0.006 | 0 |
| 31 | 1 | 0 | 0 |
| 32 | 0.996 | 0.004 | 0 |
| 33 | 0.995 | 0.005 | 0 |
| 34 | 0.712 | 0.267 | 0.021 |
| 35 | 0.234 | 0.609 | 0.157 |
| 36 | 0.001 | 0.39 | 0.609 |
| 37 | 0.001 | 0.387 | 0.612 |
| 38 | 0 | 0.045 | 0.955 |
| 39 | 0 | 0.291 | 0.709 |
| 40 | 0 | 0.411 | 0.589 |
| 41 | 0 | 0.412 | 0.588 |
| 42 | 0.22 | 0.612 | 0.168 |
| 43 | 0.503 | 0.48 | 0.017 |
| 44 | 0.984 | 0.016 | 0 |
| 45 | 0.995 | 0.005 | 0 |
| 46 | 0.996 | 0.004 | 0 |
| 47 | 1 | 0 | 0 |
| 48 | 0.371 | 0.626 | 0.003 |
| 49 | 0 | 0.002 | 0.998 |
| 50 | 0 | 0 | 1 |
| 51 | 0 | 0 | 1 |

**Table S3.** Results of discrete ancestral state reconstruction on within-individual frond dimorphism. Each species (tree tip) was categorised as being either within-individual frond monomorphic or frond polymorphic. Nodes 27 to 51 represent all internal nodes, and numbers for each state represent the probability for that node to be in that state. For the position of each internal node see Fig S3.

| Species/Node | Frond monomorphic | Frond dimorphic |
| --- | --- | --- |
| Pyrrosia_bonii | 1 | 0 |
| Pyrrosia_subfurfuracea | 1 | 0 |
| Pyrrosia_assimilis | 1 | 0 |
| Pyrrosia_lingua | 0 | 1 |
| Pyrrosia_petiolosa | 0 | 1 |
| Pyrrosia_angustissima | 1 | 0 |
| Hovenkampia_schimperiana | 1 | 0 |
| Platycerium_andinum | 0 | 1 |
| Platycerium_stemaria | 0 | 1 |
| Platycerium_angolense | 0 | 1 |
| Platycerium_ellisii | 0 | 1 |
| Platycerium_alcicorne | 0 | 1 |
| Platycerium_madagascariense | 0 | 1 |
| Platycerium_quadridichotomum | 0 | 1 |
| Platycerium_holttumii | 0 | 1 |
| Platycerium_grande | 0 | 1 |
| Platycerium_wandae | 0 | 1 |
| Platycerium_superbum | 0 | 1 |
| Platycerium_wallichii | 0 | 1 |
| Platycerium_coronarium | 0 | 1 |
| Platycerium_ridleyi | 0 | 1 |
| Platycerium_veitchii | 0 | 1 |
| Platycerium_willinckii | 0 | 1 |
| Platycerium_bifurcatum | 0 | 1 |
| Platycerium_hillii | 0 | 1 |
| Thylacopteris_papillosa | 1 | 0 |
| 27 | 1 | 0 |
| 28 | 1 | 0 |
| 29 | 1 | 0 |
| 30 | 0.99 | 0.01 |
| 31 | 0.99 | 0.01 |
| 32 | 0.95 | 0.05 |
| 33 | 0.03 | 0.97 |
| 34 | 0.75 | 0.25 |
| 35 | 0.04 | 0.96 |
| 36 | 0 | 1 |
| 37 | 0 | 1 |
| 38 | 0 | 1 |
| 39 | 0 | 1 |
| 40 | 0 | 1 |
| 41 | 0 | 1 |
| 42 | 0 | 1 |
| 43 | 0 | 1 |
| 44 | 0 | 1 |
| 45 | 0 | 1 |
| 46 | 0 | 1 |
| 47 | 0 | 1 |
| 48 | 0 | 1 |
| 49 | 0 | 1 |
| 50 | 0 | 1 |
| 51 | 0 | 1 |

**Transition matrix of discrete character evolution**

To reconstruct the origins of coloniality and morphologically differentiated individuals across the *Platycerium* phylogeny, we defined 3 possible states: Solitary (S), Colonial, monomorphic colony members (CM) and colonial, morphologically variable colony members (CP). We then built three transition matrices using the fitMK function to specify different scenarios of character evolution. The first matrix (M1) allowed all possible transitions between the states. The second (M2) only allowed unidirectional transitions, meaning only S → CM, S → CP and CM → CP were allowed. The third (M3), only allowed one-step transitions, meaning only S ⟷ CM and CM ⟷ CP were allowed. All models allowed each transition to be weighted differently based on the observed data. These matrices were chosen out of all possible ones based on biological plausibility. In particular, as all three states are observed in extant species, all matrices that did not permit to transition from S to CP (e.g. only allowing S ⟷ CM) were discarded *a priori.* The model that best fit our data was then selected using the AICc criterion and the *fitMK* function in the *phytools* package (Revell, 2012). Results are shown in Table S4.

**Table S4.** Different matrices used for the ancestral character estimation of coloniality and morphologically differentiated individuals. M3, allowing only one-step transitions, had the lowest AIC and was the best fit for our data.

| **Matrix** | **Permitted transitions** | **AIC** |
| --- | --- | --- |
| M1 | S ⟷ CM  S ⟷ CP  CM ⟷ CP | 45.140 |
| M2 | S → CM  S → CP  CM → CP | 48.427 |
| M3 | S ⟷ CM  CM ⟷ CP | 42.170 |

**Table S5** Results of Kolmogorov-Smirnov test, looking for significant differences between data simulated only from the prior distribution using the *brms* function and the actual observed data. P-values > 0.05 indicate that the prior used for the *MCMCglmm* analysis aligns with the observed data.

| **Model** | **Kolmogorov-Smirnov test** |
| --- | --- |
| Colonial ~ Strap Frond length | P = 0.72 |
| Colonial ~ Nest frond length | P = 0.69 |
| Colonial ~ Nest frond width | P = 0.71 |
| Colonial ~ Nest frond colour | P = 0.74 |
| Colonial ~ Strap frond Development | P = 0.75 |
| Colonial ~ Presence of Water storage | P = 0.64 |

**Table S6** List of all taxa included in the phylogenetic analysis of this study along with their GenBank accession numbers, following Xue et al., 2024.

| Species | Accession number |
| --- | --- |
| Platycerium alcicorne Desv. | OR601548 |
| Platycerium andinum Baker | OR601544 |
| Platycerium angolense Welw. ex Hook. | OR601546 |
| Platycerium bifurcatum (Cav.) C. Chr. | OR601559 |
| Platycerium coronarium (Mull.) Desv. | OR601553 |
| Platycerium ellisii Baker | OR601547 |
| Platycerium grande J. Sm. | OR601557 |
| Platycerium hillii T. Moore | OR601558 |
| Platycerium holttumii Joncheere & Hennipman | OR601556 |
| Platycerium madagascariense Baker | OR601550 |
| Platycerium quadridichotomum (Bonap.) Tardieu | OR601549 |
| Platycerium ridleyi Christ | OR601554 |
| Platycerium stemaria (P. Beauv.) Desv. | OR601545 |
| Platycerium superbum de Jonch. & Hennipman | OR601551 |
| Platycerium veitchii C. Chr. | OR601561 |
| Platycerium wallichii Hook. | OR601555 |
| Platycerium wandae Racib. | OR601552 |
| Platycerium willinckii T. Moore | OR601560 |
| Hovenkampia schimperiana | MW876325 |
| Pyrrosia angustissima (Giesenh. ex Diels) Tagawa & K. Iwats. | MT210543 |
| Pyrrosia assimilis (Baker) Ching | MN617019 |
| Pyrrosia bonii (Christ ex Giesenh.) Ching | NC 040226 |
| Pyrrosia lingua (Thunb.) Farw. | MT210540 |
| Pyrrosia petiolosa (Christ) Ching | MN885667 |
| Pyrrosia subfurfuracea (Hook.) Ching | NC 047436 |
| Thylacopteris papillosa (Blume) J. Sm. | MW876376 |

**Fig S1** Example of nest frond area measurement in *P. alcicorne*. Nest frond area was measured as the area inside the highlighted perimeter in ImageJ. The lowest nest frond was always considered at height = 0. The height of all other nest fronds was then measured relatively to the lowest one.

**
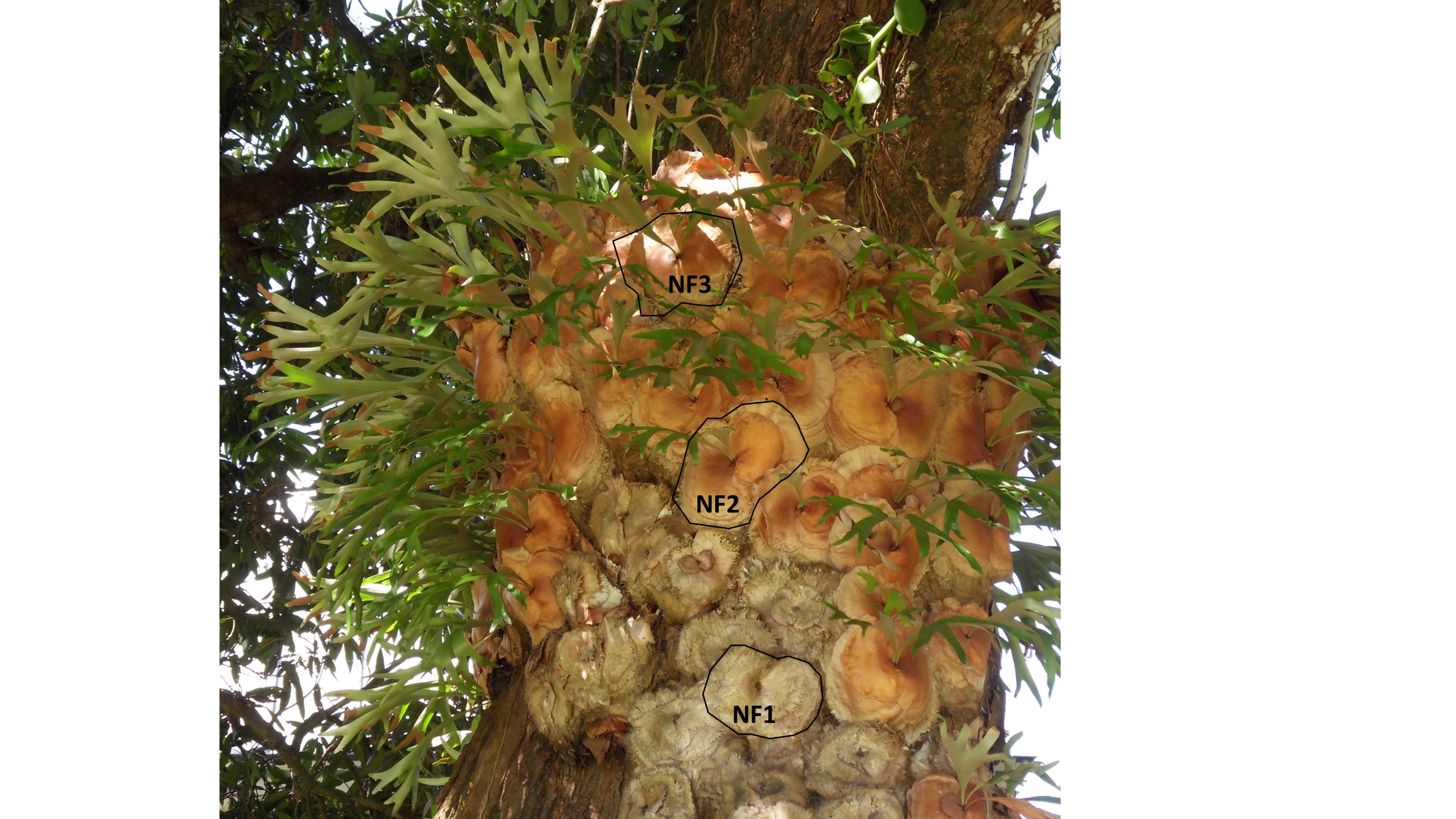
**

**Fig S2** Example of strap frond angle measurement in *P. bifurcatum*. Strap frond angle was measured as the angle created by the strap frond and the vertical axis, assuming upright vertical fronds to have an angle of 180° and vertical pendulous fronds to have an angle of 0°.

The lowest individual was always considered at height = 0. The height of all other individuals was then measured relatively to the lowest one.

**
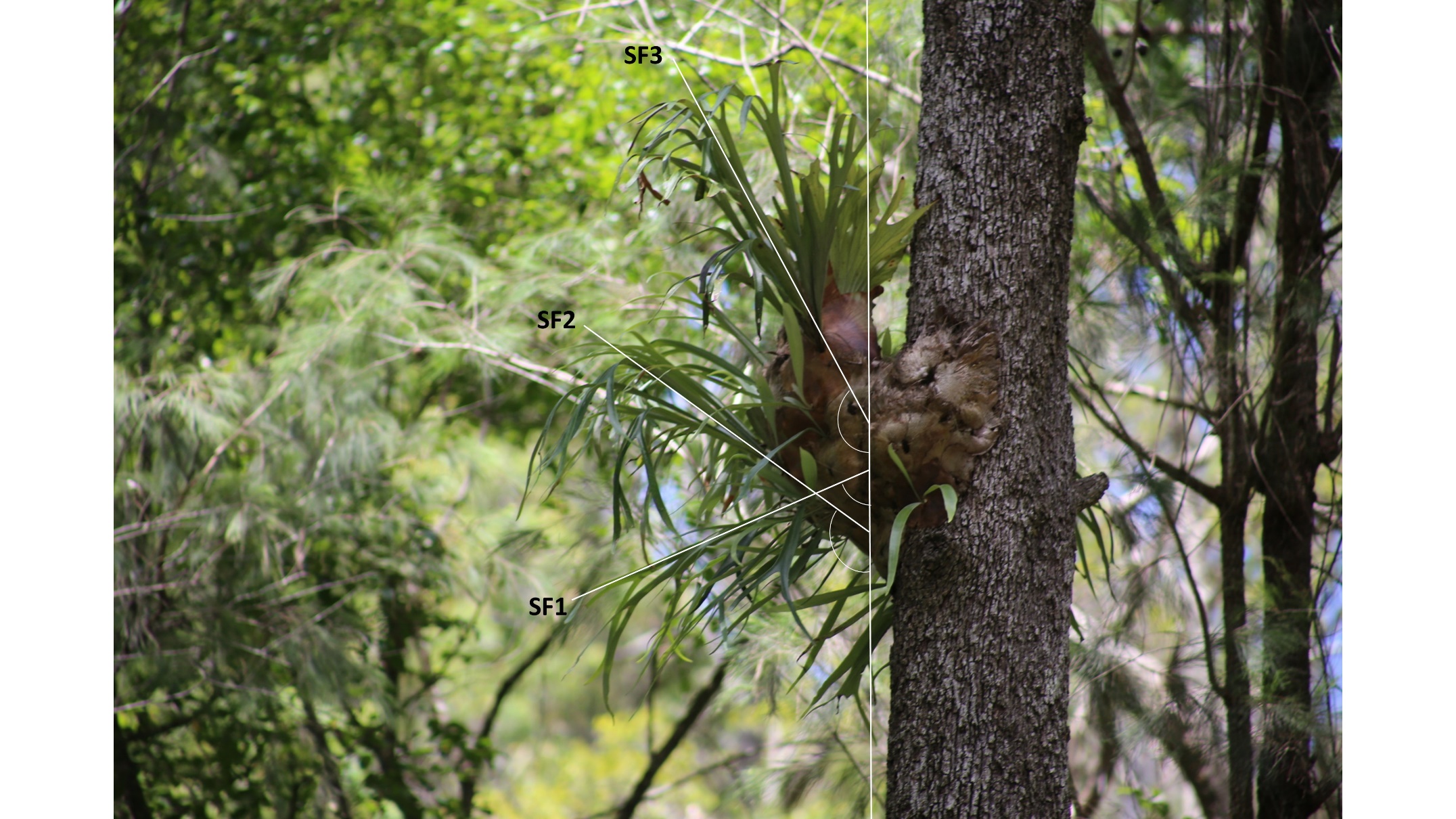
**

**Figure S3**. Maximum likelihood phylogeny of the *Platycerium* genus used for the analyses. Internal nodes are numbered. Asterisks each node indicate an ultrafast bootstrap approximation support ≥ 0.99.

**
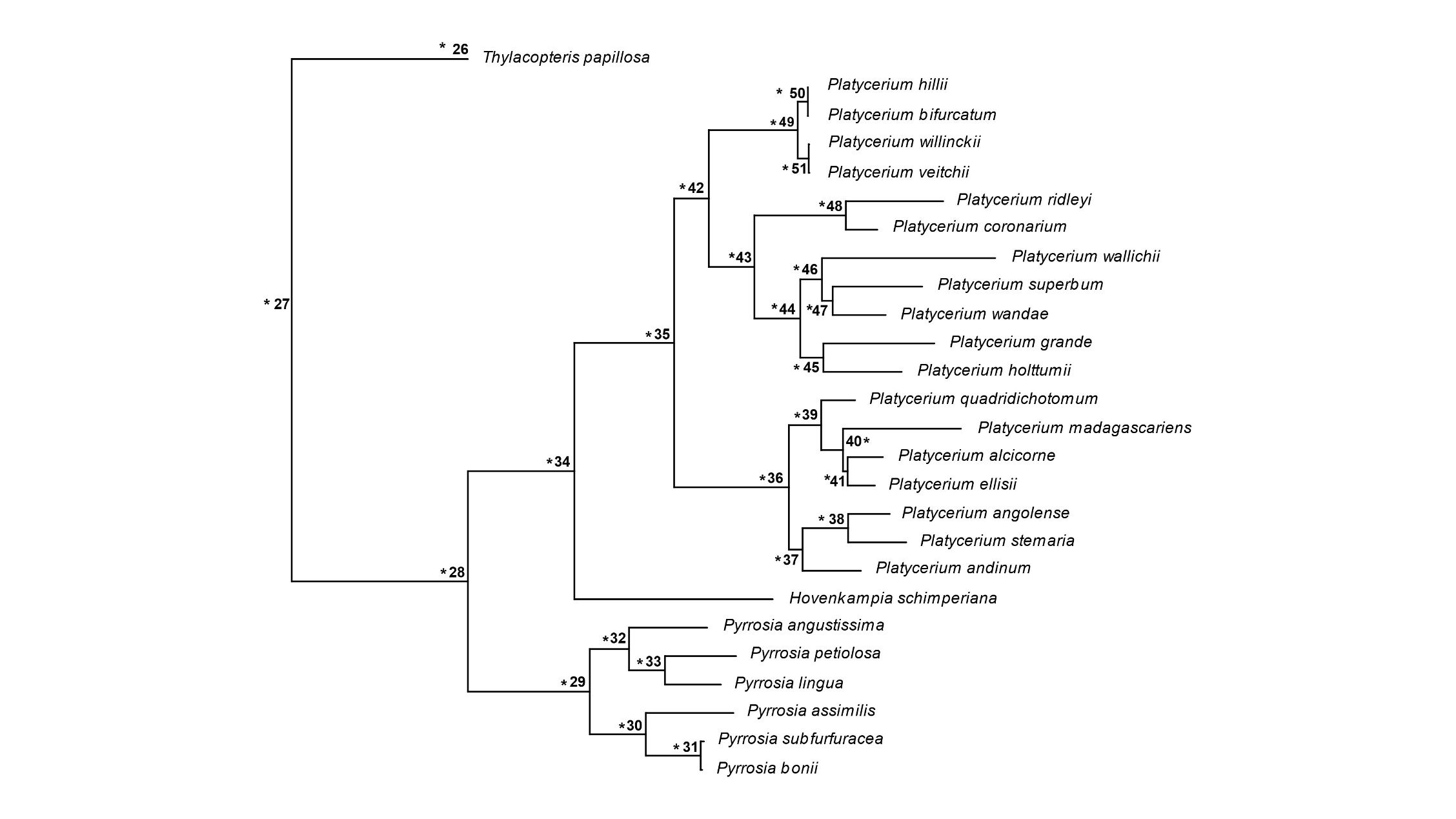
**
